# The effect of *Spirulina* sauce, as a functional food, on cardiometabolic risk factors, oxidative stress biomarkers, glycemic profile, and liver enzymes in nonalcoholic fatty liver disease patients: A randomized double‐blinded clinical trial

**DOI:** 10.1002/fsn3.2368

**Published:** 2021-06-11

**Authors:** Seyed Mohammad Mazloomi, Mohammad Samadi, Hajar Davarpanah, Siavash Babajafari, Cain C. T. Clark, Zohreh Ghaemfar, Mojtaba Rezaiyan, Abdolhamid Mosallanezhad, Maryam Shafiee, Hosein Rostami

**Affiliations:** ^1^ Nutrition Research Center Department of Food Hygiene and Quality Control School of Nutrition and Food Sciences Shiraz University of Medical Sciences Shiraz Iran; ^2^ Exercise Physiology Research Center, Life Style Institute Baqiyatallah University of Medical sciences Tehran Iran; ^3^ Nutrition Research Centre Department of Clinical Nutrition School of Food and Nutrition Sciences Shiraz University of Medical Sciences Shiraz Iran; ^4^ Centre for Intelligent Healthcare Coventry University Coventry UK; ^5^ Nephro‐Urology Research Center Shiraz University of Medical Sciences Shiraz Iran; ^6^ Health Research Center, Life Style Institute Baqiyatallah University of Medical Sciences Tehran Iran

**Keywords:** fatty liver grade, glycemic profile, inflammation, nonalcoholic fatty liver disease, *Spirulina*

## Abstract

**Objective:**

This study sought to investigate the effect of *Spirulina* on cardiometabolic risk factors, oxidative stress biomarkers, glycemic profile, and liver enzymes in nonalcoholic fatty liver disease (NAFLD) patients.

**Methods:**

This randomized, double‐blind clinical trial was performed on 46 NAFLD patients. Subjects were allocated to consume either *Spirulina* sauce or placebo, each 20 g/day for 8 weeks. Fatty liver grade, liver enzymes, anthropometric parameters, blood pressure, and serum lipids, glucose, insulin, malondialdehyde, and antioxidant capacity were assessed pre‐ and postintervention.

**Results:**

Fatty liver grade was significantly different between the two groups. A significant change for ALT (alanine aminotransferase) and AST (aspartate aminotransferase) was seen between the two groups (*p* = .03 and .02, respectively), while ALP (alkaline phosphatase) serum levels were not significantly different within or between groups. Pertaining to glycemic profile, all variables, except HOMA‐IR, were not significantly different within or between groups. Finally, statistically significant changes were seen in both MDA (malondialdehyde) and TAC (total antioxidant capacity) among the groups (*p* = .04 and <.001, respectively).

**Conclusions:**

*Spirulina* may improve fatty liver grade by modifying liver enzymes, oxidative stress, and some lipid profiles; however, there was effect of *Spirulina* on anthropometric characteristics and blood pressure.

## Graphical abstract

This study was designed to further investigate the use of spirulina in improving patients with nonalcoholic fatty liver disease (NAFLD).

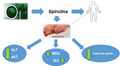

## 1 INTRODUCTION

Nonalcoholic fatty liver disease (NAFLD) is a global public health concern, with a high prevalence, impacting around 11%–16% of the population (Williams et al., 2011). NAFLD prevalence in Asia is reported to be 6%–25% (Fan, 2007) and ~2.9% in Iranian adults (Rogha et al., 2011). NAFLD is characterized by an increase in fat in the liver cells (Bellentani et al., 2000; Ma et al., 2020; Neuschwander‐Tetri & Caldwell, 2003); although it is normal for the liver to have some fat, when it exceeds 5% of liver weight, further complications are often present (Raziel et al., 2015). Indeed, the clinical importance of this disease is attributable to the liver cell apoptosis and the destruction of liver tissue, and if not diagnosed early and properly treated, irreversible cirrhosis of the liver can occur. Concomitant to NAFLD, increased blood pressure and body fat, obesity, diabetes, and insulin resistance, components of metabolic syndrome, are routinely observed (Aller et al., 2015; Ludwig et al., 1980; Raziel et al., 2015; Wang et al., 2019).

Contemporary treatment for NAFLD generally includes lifestyle modifications, particularly diet and physical activity (Rezaei et al., 2019). In this regard, functional foods have been shown to be effective in helping control metabolic dysfunction. For instance, it has been asserted that *Spirulina* intake can modify cholesterol, expand the antioxidant capacity, and decrease insulin resistance and glucose uptake (Moura et al., 2011). *Spirulina* is a multicellular, fibrous green algae found naturally in alkaline lakes. Furthermore, it is cultivated in a controlled environment for human consumption and is considered a healthy food without side effects by the United Nations Development Program (Pham et al., 2019). *Spirulina* is used as a dietary compound, due to large quantities of antioxidants (Hossain et al., 2016) such as creatine, phycocyanin (containing the triple‐pyrrole chromophore, identified as phycocyanobilin, which is covalently attached to apoprotein), minerals K, Na, Ca, Mg, Fe, and Zn, vitamins (tocopherols), eight crucial amino acids, unsaturated fatty acids, particularly linolenic acid, omega‐3 and omega‐6 fatty acids, and phenolic compounds (Ferreira‐Hermosillo et al., 2010; Moura et al., 2011; Neyrinck et al., 2017; Vázquez‐Velasco et al., 2017). Moreover, it purportedly possesses a good source of protein (containing 65–70% dry weight) (Moura et al., 2011; Neyrinck et al., 2017). Due to its anti‐inflammatory effects, *Spirulina* is consumed as a dietary supplement in many countries (Neyrinck et al., 2017). Previous studies have shown positive influences of *Spirulina* on NAFLD, oxidative stress, hyperglycemia, hypocholesterolemia, malnutrition, anemia, allergic rhinitis, cancer, substance toxicity reduction, and arterial hypertension (Fujimoto et al., 2012; Moura et al., 2011; Neyrinck et al., 2017). Indeed, *Spirulina* protects against liver toxicity in murine models by enhancing cellular antioxidant enzymes, such as superoxide dismutase, glutathione peroxidase, and catalase (Premkumar et al., 2004). Moreover, it has been confirmed in animal and human studies that protection against inflammatory diseases, including colitis and arthritis, may be conferred (Pham et al., 2019).

Therefore, due to the prevalence of NAFLD, and the relationship between fatty liver disease and high blood pressure, obesity, diabetes, and insulin resistance, concomitant to the limited number of human‐based studies in this field, we sought to investigate the effect of *Spirulina* on cardiometabolic risk factors, oxidative stress biomarkers, glycemic profile, and liver enzymes in NAFLD patients.

## 2 METHODS AND MATERIALS

### 2.1 Study design and characteristics of patients

This present study was conducted as a double‐blinded randomized controlled trial that conformed to the Declaration of Helsinki and Good Clinical Practice Guidelines. Our study protocol was reviewed and accepted by the ethics committee of Baqiyatallah University of Medical Sciences in Iran (approval number: IR.BMSU.REC. 1398.312) and enrolled in the Iranian Registry of Clinical Trials (IRCT20200304046692N1 registered on 2020‐03‐11). Eligible patients were those with NAFLD who were referred to clinical health care.

Inclusion criteria were as follows: NAFLD diagnosis by the physician (using ultrasound) and age ranges from 18 to 70 years. Individuals were not included if they were diagnosed with other diseases, such as cardiovascular disease, liver disease (cirrhosis, alcoholic liver disease, viral hepatitis, autoimmune hepatitis, biliary obstruction, primary biliary cirrhosis, and hepatic injury), cancer, renal failure, or celiac. In addition, pregnant and/or lactating women, taking drugs that cause fatty liver (methotrexate, tamoxifen, valproate, etc.), taking lipid‐lowering drugs, malnutrition that required nutritional support, following special diets such as vegetarian or raw vegetarian, and alcohol consumption were further exclusory factors. Finally, participants were excluded from the study if they did not follow the recommended treatment, were unwilling to continue the study, and had any acute diseases or infections during the study and if hospitalization occurred for period more than 5 days.

### 2.2 Sample size

The sample size was estimated to be 19 participants in each group, with a power (1−β) of 80% and *α* = 0.05, in line with a previous study (Mazokopakis et al., 2014). We estimated a 20% attrition rate, and thus, 23 patients were recruited in each group.

### 2.3 Randomization

We assigned 46 patients randomly in a 1:1 ratio, to the *Spirulina* sauce and placebo groups, respectively. To assign participants, blocked randomization with a fixed block size of 4 was used and done by a researcher who had no clinical involvement in our study.

### 2.4 Blinding

To blind patients to the samples, the sauce sachets in both groups were identical in appearance, taste, and color. The sauce sachets were coded differently in each group to blind the investigator.

### 2.5 Intervention

Participants in the *Spirulina* sauce group consumed one sachet (20 mg) of sauce containing 2 gr *Spirulina* per day for 8 consecutive weeks and similarly in the placebo group received one sachet (20 mg) of placebo per day. The sauce was produced in the Namakin factory in Shiraz, Iran. To produce the sauce, raw materials including oil, lemon juice, vinegar, salt, gum, spices, and water were used. 10% of *Spirulina* was added to the formula of sauces in the *Spirulina* sauce group, and to normalize the sensory properties, including the color of the sauce, natural dark green chlorophyll with international code E‐141 was used in the products of the placebo. The sauces were similar in terms of fat, carbohydrate, salt, flavorings, and packaging for the *Spirulina* sauce and placebo groups. The chemical composition and caloric content of the sauce are shown in Table 1.

**TABLE 1:** The chemical composition and caloric content of the sauce

Samples	Carbohydrate (%)	Protein (%)	Fat (%)	Fiber (%)	Moisture (%)	Total ash (%)	Energy (kcal/20 gr sauce)
*Spirulina* sauce	9.73	7.96	15	7.32	65.65	1.66	32.29
Placebo	9.47	0.81	15	7.06	73.54	1.18	29.57

### 2.6 Outcomes and measurement

Primary outcomes of this study were changes in the severity of fatty liver (grades 1–3), and secondary outcomes included any changes in anthropometric indices, SBP (systolic blood pressure) and DBP (diastolic blood pressure), lipid profile, HOMA‐IR, MDA, TAC, atherogenic index of plasma (AIP), and liver enzymes.

The demographic questionnaires, through face‐to‐face anamnesis interview, were completed by the main investigator. 12‐hr fasting blood samples were drawn by certified clinical staff, before and after 8 weeks. Blood samples were centrifuged, and serums were kept at −7℃ in the freezer until final measurements for lipid profile, FBS, fasting plasma insulin, MDA, TAC, AIP, and liver enzymes. Height was measured using a wall‐fixed tape to the nearest 0.1 cm. Body weight was measured to the nearest 0.1 kg using a scale, while participants were in light clothes. Standard methods were considered to measure waist circumference by a nonstretchable measured tape. BMI (body mass index) was computed as weight (kg)/height (m^2^). Blood pressure was measured twice with an interval of at least 5 min, using a mercury barometer (ALPK2), and the mean of the two measurements was considered as the subject's blood pressure. Plasma glucose levels, lipid profile, and liver enzymes were evaluated using an enzymatic colorimetric (GOD‐PAP) method (Pars Azmoon Inc.). Serum MDA and TAC amounts were assessed by enzyme‐linked immunosorbent assay (ELISA) (Diaclone).

We measured insulin sensitivity with the quantitative insulin sensitivity check index (QUICKI): QUICKI: 1/(log (fasting insulin mU/ml) × log (fasting glucose mg/dl)).

HOMA‐IR: Fasting plasma insulin (mu/ml)_fasting plasma glucose (mmol/l)/22.5.

AIP was calculated by log TG/HDL.

### 2.7 Liver ultrasound

Liver ultrasound was used for fatty liver measurement. A single expert radiologist was conducted ultrasound measurement. The grade of echogenicity, the embodiment of the diaphragm, borders of the liver vasculature, and incarnation of the posterior portion of the right hepatic lobe were used to assess fatty liver's grade (from grades 1 to 3).

### 2.8 Statistical methods

SPSS statistical software (Version 22; IBM) was used for all data analyses. The normality of the data was evaluated by the Kolmogorov–Smirnov test. We used a paired *t* test for the data with normal distribution and Wilcoxon signed‐rank test for non‐normally distributed data to evaluate variations within both groups. One‐way analysis of covariance (ANCOVA) was used for variations between groups, while a Kruskal–Wallis test was used for the data with non‐normal distribution. Statistical significance was accepted, *a* priori, at *p* < .05.

## 3 RESULTS

**FIGURE 1:**
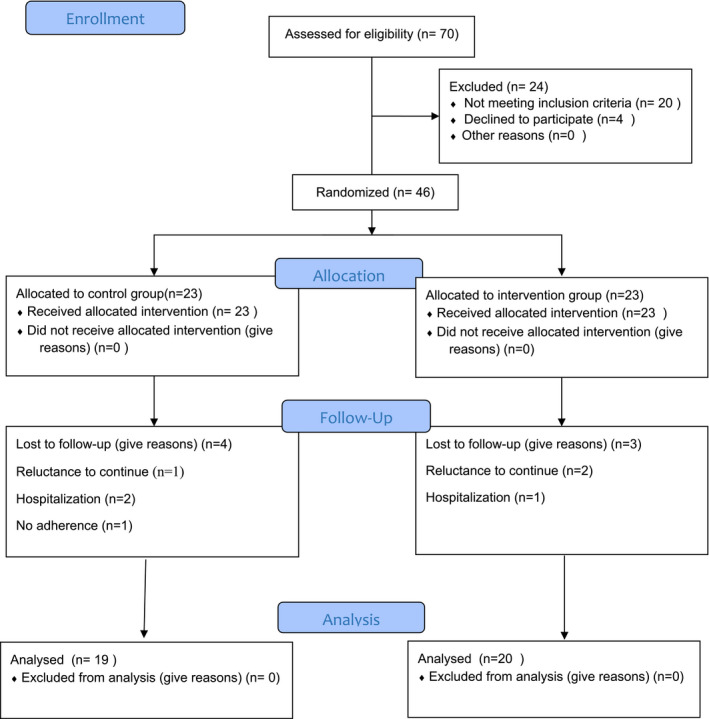
Flowchart of the trial

70 patients were assessed and finally, 46 participants completed the study. During the study, 7 participants were lost to attrition (the *Spirulina* sauce group = 3 and the placebo group = 4) because of the following reasons: reluctance to continue, hospitalization, and no adherence (Figure 1). Thus, 20 and 19 participants were analyzed in the *Spirulina* sauce and placebo groups, respectively.

### 3.1 Baseline characteristics

The baseline characteristics of the participants are illustrated in Table 2. There was no statistically significant difference between the two groups at baseline, except for MDA.

**TABLE 2:** Baseline characteristics of the participants^a^, ^b^

Variables	Placebo group (*n* = 23)	Spirulina sauce group (*n* = 23)	*p*‐value
Age, years	35.78 ± 11.14	38.87 ± 14.61	.42
Males, *n* (%)	13 (56.5)	9 (39.1)	.23
NAFLD duration, years	3.13 ± 1.57	3.56 ± 1.59	.35
Weight, kg	72.59 ± 11.87	69.34 ± 10.09	.32
BMI, kg/m^2^	25.41 ± 3.45	24.67 ± 2.75	.42
Waist circumference, cm	95.21 ± 7.10	93.78 ± 6.29	.47
ALT U/L	37.47 ± 4.37	38.86 ± 4.09	.27
AST, U/L	21.78 ± 2.66	23.13 ± 2.71	.09
ALP, U/L	40.52 ± 5.15	43.17 ± 5.73	.10
Fatty liver grade	1.82 ± 0.65	1.78 ± 0.59	.81
Fasting blood sugar, mg/dl	87.70 ± 8.63	91.43 ± 7.71	.13
Insulin, μU/ml	7.36 ± 2.95	8.30 ± 3.27	.31
Triglycerides, mg/dl	152.83 ± 21.12	165.30 ± 41.20	.20
Total cholesterol, mg/dl	185.96 ± 39.90	202.48 ± 45	.19
LDL‐C, mg/dl	117.70 ± 40.62	126.96 ± 45.18	.46
HDL‐C, mg/dl	37.61 ± 11.27	42.43 ± 8.28	.10
**MDA, μmol/L**	3.69 ± 0.51	4.04 ± 0.44	**.01**
TAC, mmol/L	0.50 ± 0.35	0.64 ± 0.26	.12
SBP, mmHg	127.91 ± 9.79	130.04 ± 7.05	.40
DBP, mmHg	91.65 ± 5.62	87.60 ± 8.02	.05

Abbreviations: ALP, alkaline phosphatase; ALT, alanine aminotransferase; AST, aspartate aminotransferase; DBP, diastolic blood pressure; HDL, high‐density lipoprotein; LDL, low‐density lipoprotein; MDA, malondialdehyde; SBP, systolic blood pressure; TAC, total antioxidant capacity.

The bold value shows a significant difference between the two groups.

^a^ Data are either means ± *SD* or *n* (%).

^b^ Qualitative variables were examined with chi‐square test; quantitative variables were tested with independent *t* test.

### 3.2 Fatty liver grade and liver enzymes

Changes in study outcomes are shown in Table 3. In comparison with the baseline, fatty liver grade decreased significantly in the *Spirulina* sauce group, concomitant to a nonsignificant increase in the placebo group. Between groups, there was a significant difference (*p* = .007).

**TABLE 3:** Fatty liver grade, liver enzymes, cardiometabolic and oxidative stress parameters, and measures before and after the intervention^a^

Variables	Week 0	Week 8	Changes	*p*‐value^b^	*p*‐value^c^	*p*‐value^e^
Fatty liver grade^d^						
Placebo group	1.82 ± 0.65	1.94 ± 0.62	0.15 ± 0.68	.33	.01	.007
*Spirulina* sauce group	1.78 ± 0.59	1.45 ± 0.51	−0.40 ± 0.75	.02		
ALT, U/L						
Placebo group	37.47 ± 4.37	36.26 ± 4.42	−0.57 ± 6.14	.68	.04	.03
*Spirulina* sauce group	38.86 ± 4.09	33.25 ± 4.52	−5.30 ± 5.41	<.001		
AST, U/L						
Placebo group	21.78 ± 2.66	21.30 ± 3.67	−0.27 ± 3.93	.76	.02	.02
*Spirulina* sauce group	23.13 ± 2.71	18.95 ± 2.72	−3.95 ± 3.70	<.001		
ALP, U/L						
Placebo group	40.52 ± 5.15	39.63 ± 4.34	−0.84 ± 6.01	.54	.50	.70
*Spirulina* sauce group	43.17 ± 5.73	40.55 ± 4.24	−2.70 ± 6.64	.08		
Triglycerides, mg/dl						
Placebo group	152.83 ± 21.12	164.89 ± 38.82	12.42 ± 25.88	.05	.04	.02
*Spirulina* sauce group	165.30 ± 41.20	138.65 ± 41.70	−30.35 ± 59.48	.03		
HDL cholesterol, mg/dl						
Placebo group	37.61 ± 11.27	38.58 ± 10.65	0.36 ± 9.1	.86	.03	.07
*Spirulina* sauce group	42.43 ± 8.29	46.40 ± 11.64	4.45 ± 7.81	.02		
Total cholesterol, mg/dl						
Placebo group	185.96 ± 39.90	195.11 ± 24.86	5.31 ± 29.6	.42	.51	.15
*Spirulina* sauce group	202.48 ± 45	186.75 ± 49.86	−13.70 ± 39.75	.14		
LDL cholesterol, mg/dl						
Placebo group	117.70 ± 40.62	123.53 ± 23.76	2.52 ± 29.46	.71	.53	.17
*Spirulina* sauce group	126.96 ± 45.18	116.60 ± 41.76	−8.10 ± 22.20	.11		
Fasting blood sugar, mg/dl						
Placebo group	87.70 ± 8.63	85.68 ± 7.71	−2.57 ± 10.84	.31	.54	.55
*Spirulina* sauce group	91.43 ± 7.71	87.20 ± 7.80	−3.6 ± 11.73	.18		
Insulin, μU/ml						
Placebo group	7.36 ± 2.95	8.21 ± 1.97	1.11 ± 2.43	.06	.37	.08
*Spirulina* sauce group	8.30 ± 3.27	7.57 ± 2.36	−0.71 ± 2.98	.29		
HOMA‐IR						
Placebo group	1.59 ± 0.66	1.75 ± 0.50	0.20 ± 0.44	.06	.49	.04
*Spirulina* sauce group	1.90 ± 0.82	1.63 ± 0.56	−0.25 ± 0.66	.10		
QUICKI						
Placebo group	0.67 ± 0.10	0.63 ± 0.06	−0.04 ± 0.07	.01	.44	.10
*Spirulina* sauce group	0.64 ± 0.11	0.65 ± 0.07	0.003 ± 0.10	.87		
Malondialdehyde, μmol/L						
Placebo group	3.69 ± 0.51	3.80 ± 0.28	0.18 ± 0.59	.19	.01	.04
*Spirulina* sauce group	4.04 ± 0.44	3.43 ± 0.51	−0.64 ± 0.74	.001		
Total antioxidant capacity, mmol/L						
Placebo group	0.50 ± 0.35	0.43 ± 0.09	−0.08 ± 0.35	.32	<.001	<.001
*Spirulina* sauce group	0.64 ± 0.26	0.79 ± 0.22	0.14 ± 0.30	.04		
Atherogenic index						
Placebo group	0.62 ± 0.14	0.63 ± 0.20	0.02 ± 0.14	.53	.01	.007
*Spirulina* sauce group	0.58 ± 0.13	0.47 ± 0.18	−0.12 ± 0.18	.007		

Abbreviations: ALP, Alkaline phosphatase; ALT, alanine aminotransferase; AST, aspartate aminotransferase; HDL, high‐density lipoprotein; HOMA‐IR, homeostasis model assessment of insulin resistance; LDL, low‐density lipoprotein; QUICKI, quantitative insulin sensitivity check index.

^a^ Data are means ± *SD*.

^b^ Paired *t* test was used for determination of the difference between baseline and endpoint values in each group.

^c^ ANOVA was used for examining the significance of the difference in changes of variables between the two groups.

^d^ Values are the mean and *SD* of fatty liver grade when it was coded as 0 (no fatty liver), 1 (mild fatty liver), 2 (moderate fatty liver), and 3 (severe fatty liver).

^e^ ANCOVA was used for examining the significance of the difference between the two groups with baseline values as a confounder.

Results indicated that serum concentration of ALT and AST was significantly reduced in the *Spirulina* sauce group, while in the placebo group, the reduction was not significant. After 8 weeks' intervention, significant changes between the two groups were seen for both of ALT and AST (*p* = .03 and .02, respectively). However, changes in ALP serum levels were not significant within or between groups.

### 3.3 Lipid profiles

In the *Spirulina* sauce group, TG serum levels decreased and HDL‐C serum levels increased significantly, in comparison with the baseline. In addition, the changes between the two groups were significant for TG (*p* = .02), and marginally insignificant for HDL‐C (*p* = .07). Changes in LDL‐C and total cholesterol serum levels did not differ within or between groups.

### 3.4 Glycemic profile

In terms of glycemic profile, there were no significant differences in FBS, insulin, and QUICKI between the *Spirulina* sauce and placebo groups, while HOMA‐IR was significantly different between groups (*p* = .04) (Table 3).

### 3.5 Oxidative stress biomarkers

In comparison with the baseline, MDA level decreased significantly in the *Spirulina* sauce group, while a significant increase was seen in TAC level. Furthermore, a significant difference was found between the two groups for MDA and TAC (*p* = .04 and <.001, respectively).

### 3.6 Atherogenic index

The atherogenic index reduced significantly in the *Spirulina* sauce group when compared to the baseline; however, no significant change was found in the placebo group. In comparison with the placebo group, changes in the *Spirulina* sauce group were significantly greater (*p* = .007).

### 3.7 Anthropometrics indexes and blood pressure

In comparison with the baseline, weight and BMI increased in the placebo group and decreased in the *Spirulina* sauce group. However, none of the changes were significant within and between groups. Similarly, the reduction in waist circumference within and between groups was not significant. Systolic and diastolic blood pressure reduced in both groups; however, this was nonsignificant within and between groups (Table 4).

**TABLE 4:** Anthropometric measures and blood pressure values during the study period^a^

Variables	Week 0	Week 8	Change	*p*‐value^b^	*p*‐value^c^	*p*‐value^d^
Weight, kg						
Placebo group	72.60 ± 11.87	73.12 ± 10.34	0.33 ± 2.48	.56	.16	.05
*Spirulina* sauce group	69.34 ± 10.09	68.54 ± 9.10	−0.65 ± 1.44	.06		
BMI, kg/m^2^						
Placebo group	24.94 ± 2.61	25.10 ± 2.52	0.16 ± 0.88	.44	.57	.10
*Spirulina* sauce group	24.82 ± 2.87	24.60 ± 2.88	−0.22 ± 0.51	.07		
Waist circumference, cm						
Placebo group	95.21 ± 7.10	94.63 ± 5.02	−0.58 ± 3.48	.47	.35	.32
*Spirulina* sauce group	93.78 ± 6.29	93.10 ± 5.11	−1.2 ± 4.67	.26		
SBP, mmHg						
Placebo group	127.91 ± 9.79	126 ± 7.37	−2.89 ± 7.85	.12	.68	.81
*Spirulina* sauce group	130.04 ± 7.05	126.95 ± 7.12	−3.15 ± 9.18	.14		
DBP, mmHg						
Placebo group	91.65 ± 5.62	88.68 ± 5.40	−2 ± 6.06	.16	.06	.18
*Spirulina* sauce group	87.60 ± 8.02	85.05 ± 6.07	−2.2 ± 7.15	.17		

^a^ Data are means ± *SD*.

^b^ Paired *t* test was used for determination of the difference between baseline and endpoint values in each group.

^c^ ANOVA was used for examining the significance of the difference in changes of variables between the two groups. BMI, body mass index; SBP, systolic blood pressure; and DBP, diastolic blood pressure.

^d^ ANCOVA was used for examining the significance of the difference between the two groups with baseline values as a confounder.

### 3.8 Dietary intakes and Physical activity

Energy and macronutrients' dietary intakes were not significantly different between groups during the intervention (Table 5). Similarly, physical activity did not show significant change between two groups.

**TABLE 5:** Dietary intakes and physical activity of the participants during the intervention^a^

Variables	Week 0	Week 8	Change	*p*‐value^b^	*p*‐value^c^	*p*‐value^d^
Energy, kcal/day						
Placebo group	2,386.76 ± 482.59	2,374.91 ± 459.52	−19.10 ± 98.79	.41	.13	.41
*Spirulina* sauce group	2,240.02 ± 407.09	21.68.51 ± 383.18	−61.32 ± 145.11	.07		
Carbohydrate, g/day						
Placebo group	328.17 ± 66.35	325.66 ± 62.61	−3.51 ± 14.25	.30	.14	.24
*Spirulina* sauce group	308 ± 55.97	297.81 ± 53	−8.79 ± 18.83	.05		
Protein, g/day						
Placebo group	89.50 ± 18.09	88.69 ± 16.98	−1.08 ± 4.19	.27	.34	.10
*Spirulina* sauce group	82 ± 14.75	83.77 ± 14.84	2.45 ± 5.44	.06		
Fat, g/day						
Placebo group	79.55 ± 16.08	78.64 ± 15.20	−1.16 ± 4	.22	.16	.93
*Spirulina* sauce group	74.66 ± 13.56	72.34 ± 12.37	−1.97 ± 4.99	.09		
MUFA, g/day						
Placebo group	19.89 ± 3.38	19.08 ± 4.59	−1.01 ± 6.22	.48	.12	.30
*Spirulina* sauce group	20 ± 4.53	21.25 ± 3.94	1.30 ± 2.87	.06		
PUFA, g/day						
Placebo group	18.68 ± 4.07	17.18 ± 3.84	−1.70 ± 4.42	.11	.10	.72
*Spirulina* sauce group	16.27 ± 3.99	18 ± 4.31	1.59 ± 3.89	.08		
SFA, g/day						
Placebo group	14.87 ± 3.67	12.70 ± 4.48	−1.94 ± 5.62	.14	.33	.06
*Spirulina* sauce group	13.48 ± 3.03	11.43 ± 3.55	−2.24 ± 5.73	.09		
Fiber, g/day						
Placebo group	47.08 ± 21.10	47.68 ± 18.64	1.40 ± 16.99	.72	.26	.54
*Spirulina* sauce group	42.25 ± 15.35	41.37 ± 16	1.14 ± 15.49	.74		
Physical activity, Met‐min/weeks						
Placebo group	685.30 ± 302.35	645.68 ± 249.27	−44.10 ± 157.66	.23	.06	.72
*Spirulina* sauce group	896.17 ± 434.185	869.50 ± 439.80	−80.45 ± 284.18	.22		

Abbreviations: MUFA, monounsaturated fatty acids; PUFA, polyunsaturated fatty acids; SFA, saturated fatty acids.

^a^ Data are means ± *SD*.

^b^ Paired *t* test was used for determination of the difference between baseline and endpoint values in each group.

^c^ ANOVA was used for examining the significance of the difference in changes of variables between the two groups.

^d^ ANCOVA was used for examining the significance of the difference between the two groups with baseline values as a confounder.

There were no significant correlations between MDA and fatty liver grade, ALT, AST, Insulin, and HOMA‐IR, where all correlation coefficients and accompanying *p*‐values are shown in Table 6.

**TABLE 6:** Correlation analysis between MDA with fatty liver grade, ALT, AST, insulin, and HOMA‐IR in patients with nonalcoholic fatty liver disease

Variables	Placebo group		*Spirulina* sauce group	
	MDA		MDA	
	Correlation coefficients (*r*)	*p*‐value	Correlation coefficients (*r*)	*p*‐value
Fatty liver grade	.41	.08	−.16	.49
ALT, U/L	−.32	.18	.08	.71
AST, U/L	−.18	.45	−.19	.43
Insulin, μU/ml	.30	.22	−.02	.94
HOMA‐IR	.29	.23	−.04	.87

Note

The correlation between data was examined using partial correlation by controlling MDA in baseline.

*p*‐values of <.05 were considered to indicate significance.

Abbreviations: ALT, alanine aminotransferase; AST, aspartate aminotransferase; HOMA‐IR, homeostasis model assessment of insulin resistance; MDA, malondialdehyde

## 4 DISCUSSION

To the authors' knowledge, this study represents one of the first randomized double‐blinded clinical trial human studies to have evaluated the effect of *Spirulina* in NAFLD patients. Our results revealed that 8 weeks' consumption of *Spirulina* can improve fatty liver grade, oxidative stress markers, TG, and HOMA‐IR index. However, no improvement was found in FBS, insulin, QUICKI, blood pressure, and anthropometric indices.

Fatty liver grade decreased significantly in the *Spirulina* group in comparison with the placebo group, while a significant change in ALT and AST was seen between the two groups. While ALP serum levels were not significantly different within or between groups. To support the hepatoprotective effect of *Spirulina*, one animal study evaluated the capability of *Spirulina maxima* to inhibit fatty liver development, which is induced in rats by a single dose of intraperitoneal carbon tetrachloride (1 ml/kg). This study demonstrated that AST and liver triacylglycerides reduced, and liver cholesterol did not increase, after carbon tetrachloride treatment in rats fed on a diet with *Spirulina* (Torres‐Durán et al., 1998). Another animal study also showcased inhibitory effects of *Spirulina maxima* on fatty liver exacerbation (Blé‐Castillo et al., 2002). A human‐based case study, conducted in three Mexican NAFLD patients, also showed a significant reduction in ALT blood level concomitant to ameliorating dyslipidemia (Ferreira‐Hermosillo et al., 2010). Indeed, further support for the hepatoprotective effect of *Spirulina* has been shown by the reduction of aminotransferases in NAFLD patients (Moura et al., 2011). The available animal data and few human studies have posited that the hepatoprotective effects of *Spirulina* are mainly attributed to the C‐phycocyanin, β‐carotene, and vitamin E content, eliciting antioxidant and anti‐inflammatory effects (Blé‐Castillo et al., 2002; Browning et al., 2006; Han et al., 2006; Mazokopakis et al., 2014; Nagaoka et al., 2005; Neyrinck et al., 2017; Ng et al., 2007; Nseir et al., 2012; Premkumar et al., 2004; Serban et al., 2016; Torres‐Durán et al., 1998; Westerbacka et al., 2005). Other components of *Spirulina*, such as gamma‐linolenic acid, selenium, and chlorophyll, have been shown to have a potential hepatoprotective roles (Torres‐Durán et al., 1998). The antioxidant and anti‐inflammatory property of *Spirulina*, and its constituents, might contribute to a reduction in inflammatory cytokines such as interleukin‐6 (IL‐6) and tumor necrosis factor‐α (TNF‐α) and an increase in anti‐inflammatory cytokines such as adiponectin, indicating an improvement in oxidative stress, in NAFLD patients (Blé‐Castillo et al., 2002; Browning et al., 2006; Samuels et al., 2002). Additionally, *Spirulina* is reportedly able to modulate the gut microbiota and to activate the immune system, a mechanism that may be involved in the improvement of hepatic inflammation (Moura et al., 2011).

Although our study revealed a significant improvement in MDA, there was no significant correlation between MDA with fatty liver grade and liver enzymes. Comparing the differences in both MDA and TAC between the groups, significant changes were evident. Concordant with our study, evaluating the effect of *Spirulina,* for 45 days, on liver‐injured mice, demonstrated a reduction of MDA and ALT and an increase of superoxide dismutase (SOD) activity (Ding et al., 2004). Moreover, these outcomes are similar to the findings of Lu et al., who assessed the effects of 3‐week *Spirulina* supplementation on preventing skeletal muscle damage. Lu et al. indicated that MDA was significantly reduced, while the activity of SOD and glutathione peroxidase (GP *_x_* ) was significantly increased after consumption of *Spirulina* supplementation (Lu et al., 2006). It has been shown that *Spirulina* can increase antioxidant enzymes such as superoxide dismutase, catalase, and glutathione peroxidase (Neyrinck et al., 2017). The antioxidant properties of *Spirulina* species have received wide attention in recent decades and have been supported by numerous in vitro and in vivo studies (Abdel‐Daim et al., 2013; Upasani & Balaraman, 2003). Phycocyanin, regarded as the major antioxidant in *Spirulina*, is a free radical scavengers (hydroxyl, alkoxyl, and peroxyl radicals) and contributes to the downregulation of iNOS (inducible nitric oxide synthase) and subsequent reduction in nitrite production and lipid peroxidation prevention (Deng & Chow, 2010; Gutiérrez‐Rebolledo et al., 2015; Riss et al., 2007). While in Qing et al., SOD and CAT (catalase) were the antioxidant enzymes reportedly contained within *Spirulina maxima* (Qing et al., 2003). Other antioxidant enzymes found in *Spirulina* include total GPx, GPx‐Se, and GR (glutathione reductase), which are posited to remain active and ameliorate cellular glutathione increases (Bermejo et al., 2008).

Although, in the resent study, TG levels were improved, there were no significant improvements in HDL‐C, LDL‐C, and total cholesterol levels. Furthermore, the atherogenic index reduced significantly in the *Spirulina* group when compared with the placebo group. Samuels et al. reported protective effects of 1 g/day *Spirulina* on lipid profile in patients with hyperlipidemic nephrotic syndrome (Samuels et al., 2002). Further, in Nagakoa et al., an inhibitory effect of *Spirulina platensis* on jejunal cholesterol absorption was found (Nagaoka et al., 2005). According to Li‐Kun H et al., an active component of *Spirulina*, glycolipid H‐b2, may be responsible for triacylglyceride reduction in a dose‐dependent manner, while phycocyanin may be responsible for the hypocholesterolemic effects of *Spirulina* via pancreatic lipase inhibition (Han et al., 2006). Although the exact mechanism for the apparent hypolipidemic effects of *Spirulina* is not fully understood (Nagaoka et al., 2005), it has been claimed that the C‐phycocyanin protein, a main ingredient of *Spirulina*, has the potential to improve lipid profile by enhancing glutathione peroxidase and superoxide dismutase activity, free radical scavenging, downregulating NADPH (nicotinamide adenine dinucleotide phosphate), oxidase expression, and lipid peroxidation. Furthermore, reductions in cholesterol and bile acid reabsorption have been suggested (Sharma et al., 2011; Upasani & Balaraman, 2003). Glycolipid H‐b2 and phycocyanin, other ingredients of *Spirulina*, can, reportedly, prevent pancreatic lipase activity, while its gamma‐linolenic acid (GLA), α‐linolenic acid, linolenic acid, and niacin contents have a role in the regulation of cholesterol synthesis and hypolipidemic effects of *Spirulina* (Han et al., 2006; Mazokopakis et al., 2014). Furthermore, the activity of lipoprotein lipase and hepatic triglyceride lipase is shown to be increased following *Spirulina* supplementation (Serban et al., 2016). Another protective effect of *Spirulina* arises from its specific low‐calorie, low‐fat, cholesterol‐free, and high‐protein nature (Browning et al., 2006; Westerbacka et al., 2005), which might contribute to weight loss and thus support a reduction in TG levels and decelerate the HDL apoA‐I catabolism, with a simultaneous decline in the secretion of HDL apoA‐I (Han et al., 2006; Nagaoka et al., 2005; Ng et al., 2007; Nseir et al., 2012).

In terms of glycemic profile, HOMA‐IR decreased significantly, and while FBS and insulin decreased, these changes were not statistically significant. In Parikh et al., 2 g/day *Spirulina* supplementation for 8 weeks revealed a statistically significant effect of *Spirulina* on reducing fasting blood glucose and postprandial blood glucose levels as well as HbA_1c_ level (Parikh et al., 2001). Some other studies have also shown that *Spirulina* consumption could reduce blood glucose levels (Aissaoui et al., 2017; Huang et al., 2018). *Spirulina* can putatively reduce blood glucose levels by stimulating β cell activity and increasing glucose transport to peripheral tissues (Layam & Reddy, 2006); moreover, it has been posited that the hypoglycemic effect of *Spirulina* might arise from its fiber content, creating less glucose absorption, and the high‐quality protein in *Spirulina,* which can generate peptides and polypeptides and trigger insulin secretion (Hernández‐Alonso et al., 2017), thus maintaining the plasma glucose level.

In our study, there was no significant improvement in anthropometric indices. Indeed, there are limited studies regarding the effects of *Spirulina* on weight loss (Mazokopakis et al., 2014). However, discordant with our results, another study found *spirulina* could elicit positive effects on body weight, which might be owing to the relatively higher dose and a longer period of intervention (6 g/day for 6 months in comparison with 1 g/day for 3 months in our study). The potential effect of *Spirulina* on weight loss might be attributable to its low‐fat and low‐carbohydrate content (Mazokopakis et al., 2014). Additionally, *Spirulina* is a source of phenylalanine, a potent releaser of cholecystokinin, which might help to regulate the brain's appetite center (Ballinger & Clark, 1994; Gibbs et al., 1976; Silverstone & Goodall, 1984).

Although the effect of *Spirulina maxima* in the inhibition of synthesis and releasing of arachidonic acid, and its vasoconstricting metabolites, and increasing nitric oxide synthesis (a well‐known vasodilatation metabolite) is shown in some studies (Mascher et al., 2006), another potential mechanism to ameliorate blood pressure increases might be attributed to the inhibition of platelet aggregation, anti‐inflammatory effects, and the high potassium and low sodium contents of *Spirulina* (Guan et al., 2007; Hsiao et al., 2005). In our study, we did not find any significant ameliorating effects of *Spirulina* on blood pressure; however, this might be due to the normal baseline blood pressure levels and weight of our participants.

### 4.1 Strengths and limitations

This study represents one of the first randomized double‐blinded clinical trial human studies to have evaluated the effect of *Spirulina* in NAFLD patients. In addition, potential errors in grading fatty liver were decreased due to the radiologist being blinded to the group allocation of the patients. However, despite the strength and novelty of our study, several limitations must be considered. For instance, a relatively short period of intervention and a low sample size conceivably hindered the identification of effects of *Spirulina* in some parameters; while a lack of measurement of inflammatory factors could have further impacted the overall results. Therefore, in light of the aforementioned strengths and limitations, further studies with longer intervention duration, larger sample size, and additional evaluation of inflammatory factors are suggested.

## 5 CONCLUSION

We found that 8 weeks' supplementation of *Spirulina* improved fatty liver grade, oxidative stress markers, TG, and HOMA‐IR; however, no significant change was found in blood pressure and anthropometric indices.

## CONFLICT OF INTEREST

All authors declare that they have no conflict of interest.

## ETHICAL APPROVAL

Also, our study conformed to the Declaration of Helsinki and Good Clinical Practice Guidelines and study protocol was reviewed and accepted by the ethics committee of Baqiyatallah University of Medical Sciences in Iran (approval number: IR.BMSU.REC. 1398.312) and enrolled in the Iranian Registry of Clinical Trials (IRCT20200304046692N1 registered on 2020‐03‐11).
