# EXPRESSION OF CONCERN: The Effect of Spirulina Sauce, as a Functional Food, on Cardiometabolic Risk Factors, Oxidative Stress Biomarkers, Glycemic Profile, and Liver Enzymes in Nonalcoholic Fatty Liver Disease Patients: A Randomized Double‐blinded Clinical Trial

**DOI:** 10.1002/fsn3.70006

**Published:** 2025-02-02

**EXPRESSION OF CONCERN**: S. M. Mazloomi, M. Samadi, H. Davarpanah, S. Babajafari, C. C. T. Clark, Z. Ghaemfar, M. Rezaiyan, A. Mosallanezhad, M. Shafiee, and H. Rostami, “The Effect of Spirulina Sauce, as a Functional Food, on Cardiometabolic Risk Factors, Oxidative Stress Biomarkers, Glycemic Profile, and Liver Enzymes in Nonalcoholic Fatty Liver Disease Patients: A Randomized Double‐blinded Clinical Trial,” *Food Science & Nutrition* 10, no. 2 (2022): 317–328, https://doi.org/10.1002/fsn3.2368.

This Expression of Concern is for the above article, published online on 11 June 2021 in Wiley Online Library (wileyonlinelibrary.com), and has been issued by agreement between the journal Editor‐in‐Chief, Martin Lo; and Wiley Periodicals LLC. The Expression of Concern has been agreed due to concerns raised by a third party on the data presented in the article. The authors collaborated on the investigation and provided the raw data underlying the study. An expert statistical assessment of the data indicated that the dietary intake information appears implausible. However, since the source of the experimental error causing these issues remains unclear and the identified concerns do not undermine the overall conclusions of the article, the journal has decided to issue an Expression of Concern to inform and alert readers to disregard any statements related to the dietary intake data.

